# Isolation of *Sphingomonas paucimobilis* from an ocular infection and identification using ribosomal RNA gene: First case report from Iran

**DOI:** 10.1002/ccr3.7715

**Published:** 2023-07-18

**Authors:** Fatemeh Zeynali Kelishomi, Faezeh Mohammadi, Mohaddeseh Khakpoor, Rasoul Malekmohammadi, Farhad Nikkhahi

**Affiliations:** ^1^ Department of Microbiology, School of Medicine Qazvin University of Medical Sciences Qazvin Iran; ^2^ Medical Microbiology Research Center Qazvin University of Medical Sciences Qazvin Iran

**Keywords:** 16sr RNA sequencing, antibiotic resistance, case report, ocular infection, *Sphingomonas paucimobilis*

## Abstract

**Key Clinical Message:**

*Sphingomonas paucimobilis* can cause infection in healthy people. As this bacterium is slow‐growing, special attention should be paid to the timely diagnosis and control of its antibiotic resistance to prevent the spread of resistant strains.

**Abstract:**

This study reports a case of ocular infection caused by *Sphingomonas paucimobilis* and its treatment with various antibiotics. A middle‐aged woman with prolonged purulent eye discharge was admitted to an ophthalmology clinic in Qazvin, Iran. A strain of *S. paucimobilis* was isolated from the patient. The sample was identified by Sanger sequencing of the 16s rRNA gene, and an antibiogram test was performed to determine its resistance profile. The patient was treated with ceftazidime and levofloxacin eye drops. The bacterial culture was negative 18 days after starting ceftazidime and levofloxacin treatment. The antibiogram results showed that the isolated bacterium was resistant to aminoglycosides and colistin. This study highlights that *S. paucimobilis* can cause disease even in immunocompetent individuals. Due to the different resistance profiles of this bacterium, treatment should be based on antibiogram results.

## Graphical abstract

A direct smear of the patient’s eye discharge showed the presence of gram‐negative nonspore‐forming bacilli. Further results showed that these bacilli were *Sphingomonas paucimobilis*.

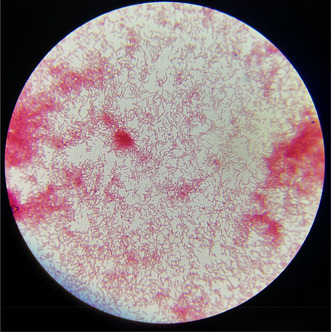

## 1 BACKGROUND

*Sphingomonas paucimobilis* is a gram‐negative, glucose‐non‐fermenting, strictly aerobic bacillus that is motile and has a polar flagellum. The bacterium is widely distributed in the environment and is responsible for hospital‐ and community‐acquired infections. This bacterium causes diseases in both immunocompromised and immunocompetent individuals. Although this bacterium is rarely isolated from clinical samples, reports of its isolation have increased in recent years.^1^ Therefore, it is necessary that its timely diagnosis and treatment be placed on the agenda of laboratories and ophthalmology clinics.

## 2 CASE PRESENTATION

**FIGURE 1:**
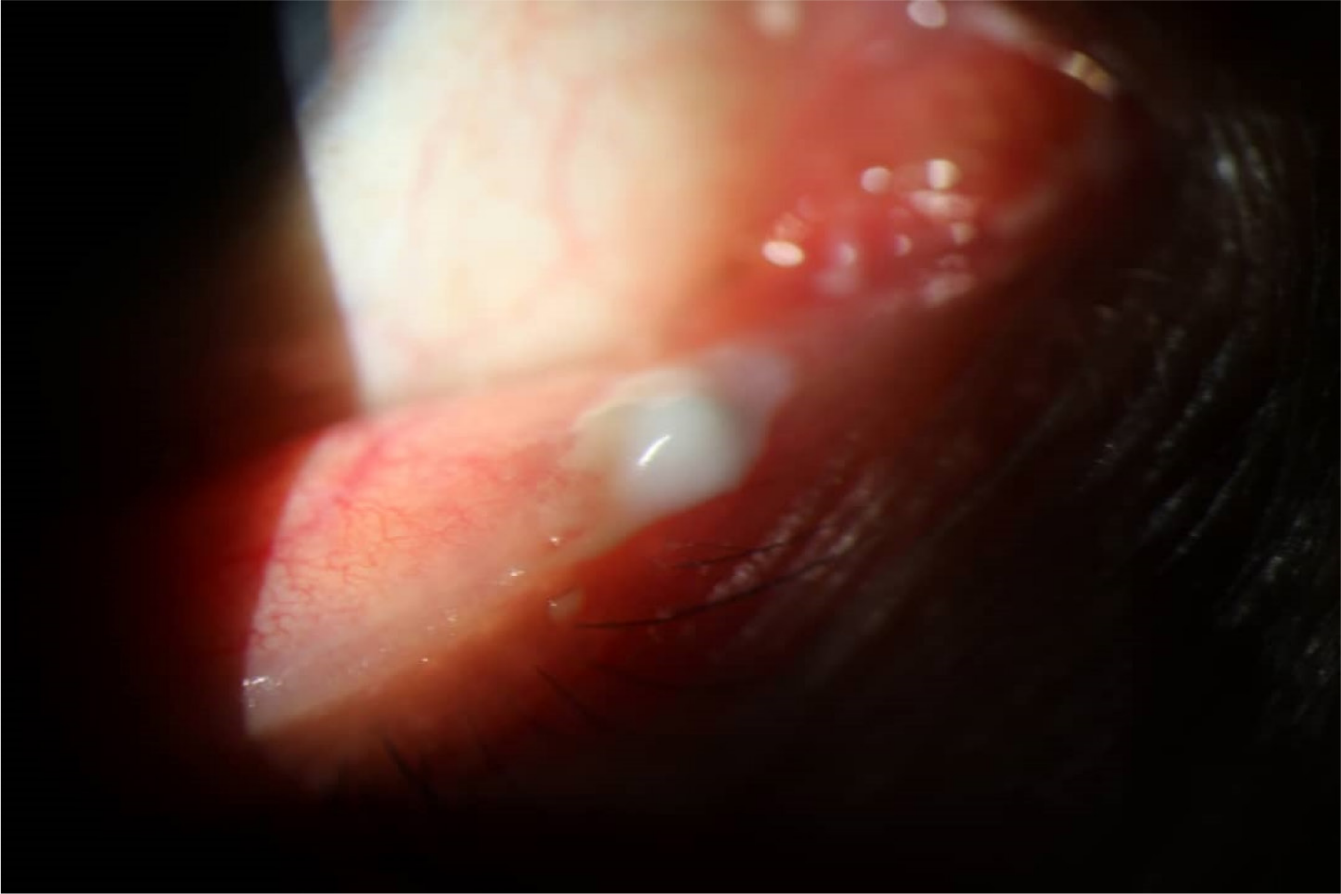
The photograph of the right eye shows severe redness and semidry purulent discharge.

A 34‐year‐old woman who complained of eye redness, irritation, and purulent discharge from her right eye for 8 days was admitted to a private ophthalmology clinic in Qazvin, Iran. The patient did not mention any history of having similar clinical conditions, trauma, and medical examinations in and around the eye. Also, the patient had no history of taking any topical or systemic medications. In the initial visit (and during treatment), the patient was not in pain. Eye examination revealed a positive papillary reaction in the lower punctum, and severe redness and irritation. Semidry and turbid purulent discharge was observed at the site of infection. Considering that the patient was not immunocompromised, eyelid and lacrimal sac massage was performed to detect the infection in the lacrimal sac. Discharge increased with lower eyelid massage (Figure 1). The patient had no significant medical history, and her blood sugar level was within normal range. The treatment was empirically initiated with eye massage, gentamicin 0.3% (dose: 3 mg/mL‐every 12 h, dosage: 6 mg/day), and sulfacetamide 10% (dose: 100 mg/mL‐every 8 h, dosage: 300 mg/day). The patient was told to avoid touching the eyes, sharing her personal items such as makeup and eyeglasses with others, and wearing contact lenses and to take her medications as prescribed. Third days after the patient visited the clinic, discharge and redness persisted, but the irritation resolved.

After 6 days with no response to treatment, increased eye discharge, and persistent redness, considering the lack of bulging in the lacrimal sac, her physician suspected canaliculitis caused by *Actinomyces israelii*. Therefore, canaliculotomy was performed, and a sample of eye discharge was sent to the Microbiology Department of the Qazvin University of Medical Sciences laboratory for direct smear and culture.

**FIGURE 2:**
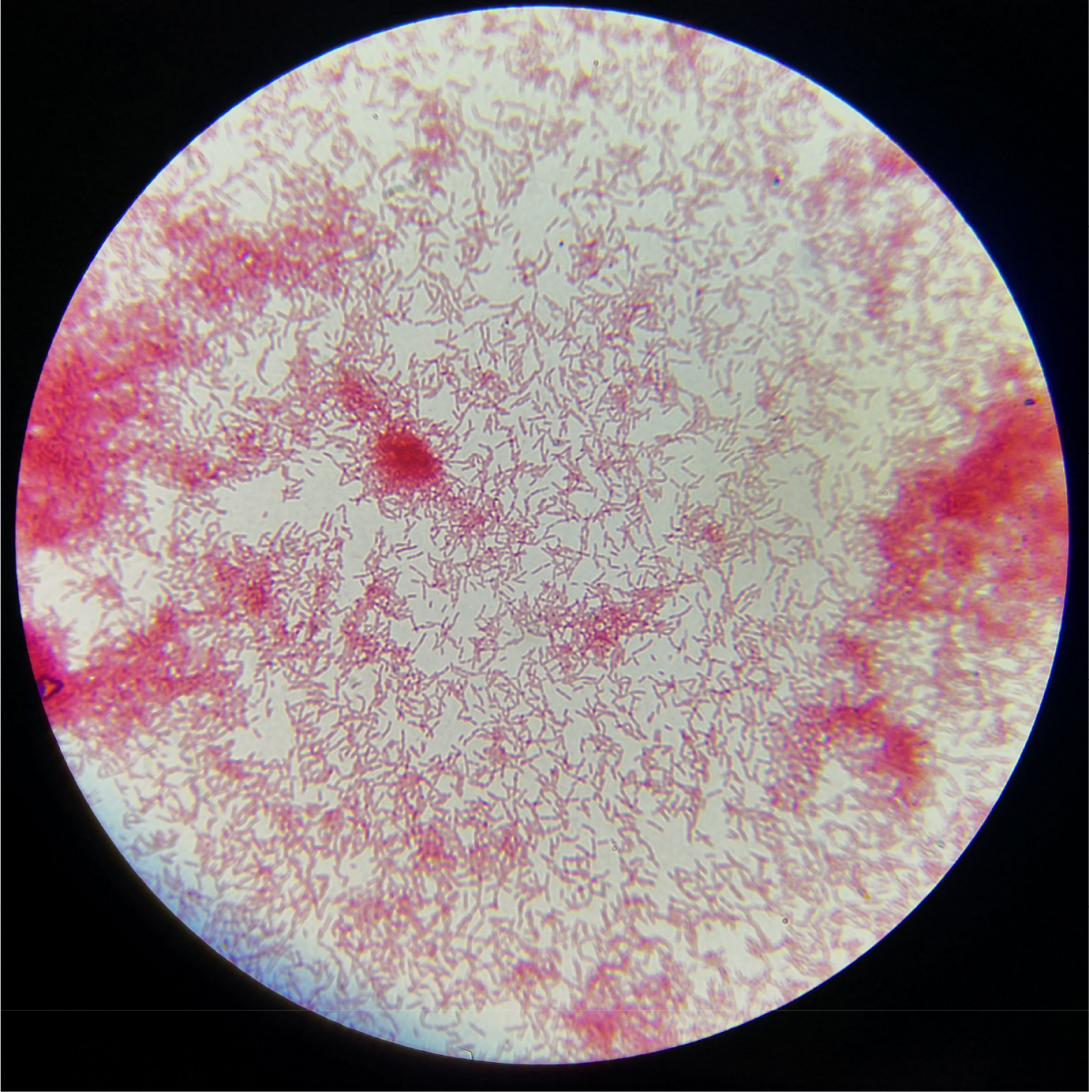
Gram‐negative bacilli in direct smear (×400).

Gram‐negative nonspore‐forming bacilli have been reported in direct smears (Figure 2). Therefore, gram‐negative bacilli were treated experimentally with ceftazidime 5% (dose: 50 mg/mL‐every 8 h, dosage: 150 mg/day) and levofloxacin 0.5% (dose: 5 mg/mL‐every 4 h, dosage: 30 mg/day) eye drops.

**FIGURE 3:**
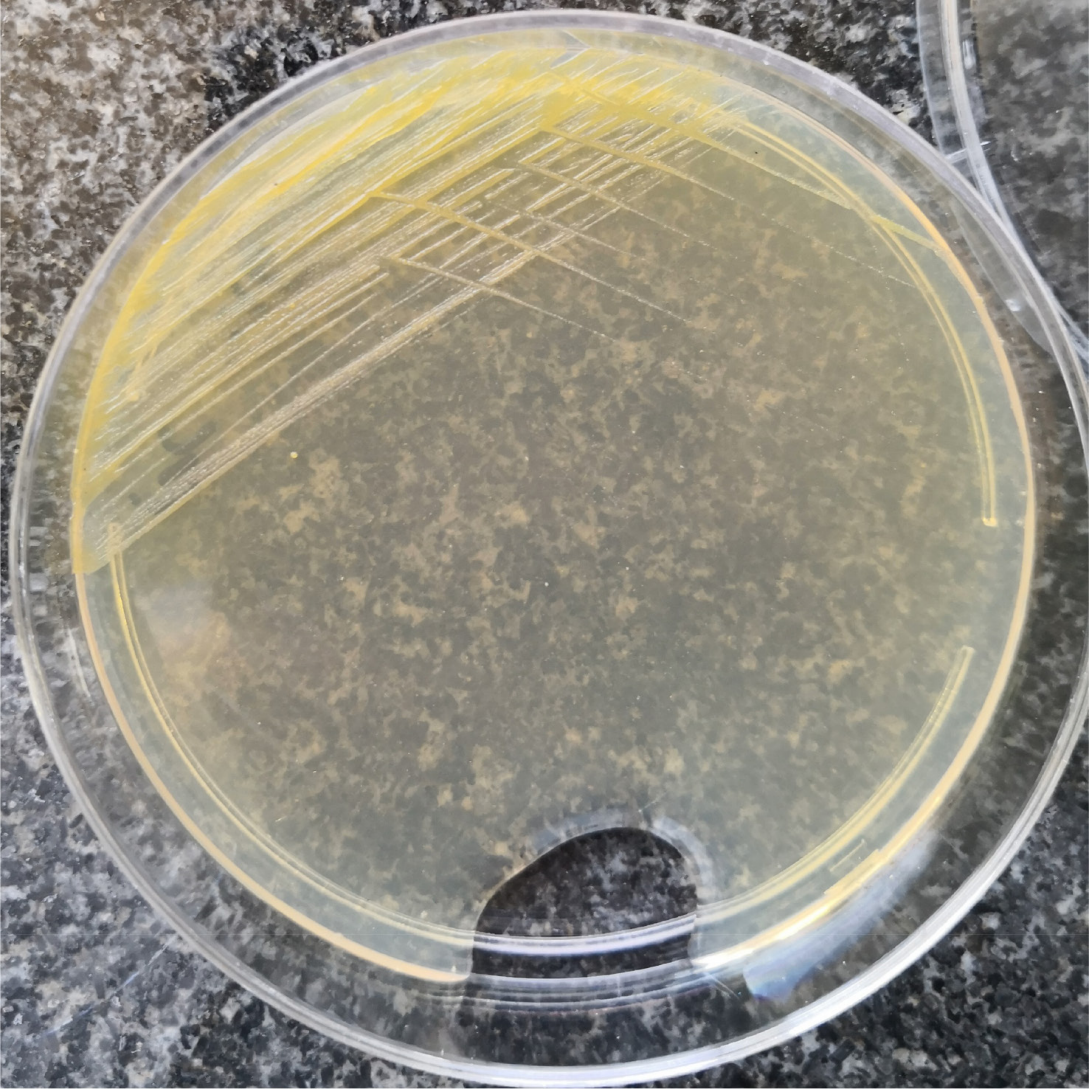
Bacterial growth on nutrient agar after 3 days of incubation.

The sample was cultured on sheep blood agar, nutrient agar, chocolate agar, and McConkey agar (Liofilchem). The culture medium was incubated for 5 days under aerobic conditions. The bacterial culture results showed that this bacterium was slow‐growing, with yellow‐pigmented colonies appearing on the third day of incubation (Figure 3). The catalase and oxidase tests were positive.

Due to the lack of standard criteria for the biochemical detection of this bacterium, and the lack of access to MALDI‐TOF and Vitek systems, polymerase chain reaction (PCR) was performed for the 16s rRNA gene, and the amplified PCR product was sent to Macrogen, Korea for Sanger sequencing. Sequencing results showed that the target bacterium was *S. paucimobilis*.

Antibiogram tests were performed using the disk diffusion method and Muller Hinton Agar (Liofilchem, Italy) for the antibiotics ceftazidime, cefotaxime, cefepime, piperacillin/tazobactam, aztreonam, imipenem, meropenem, trimethoprim‐sulfamethoxazole, tigecycline, ciprofloxacin, levofloxacin, amikacin, and gentamicin (Mast Group Ltd, United Kingdom). Resistance to colistin was assessed using the microbroth dilution method. As there were no guidelines in the CLSI (Clinical and Laboratory Standard Institute) and EUCAST (European Committee on Antimicrobial Susceptibility Testing) for interpreting *S. paucimobilis* antibiogram results, we used the “Other Non‐Enterobacterals” category in the CLSI, and the “*Acinetobacter baumannii*” in EUCAST as criteria for interpreting our disk diffusion and MIC (Minimum Inhibitory Concentration) results (MIC >2 μg/mL were considered resistant).^2^, ^3^

The antibiogram results showed sensitivity to ceftazidime, cefotaxime, cefepime, aztreonam, imipenem, meropenem, ciprofloxacin, and levofloxacin. Resistance to piperacillin/tazobactam, trimethoprim‐sulfamethoxazole, amikacin, gentamicin, tigecycline, and colistin was also observed.

Based on the antibiogram results, antibiotics prescribed by the ophthalmologist were deemed appropriate for treatment. The follow‐up of the patient was done in the form of an examination with torch, checking visual acuity, and checking the presence or absence of initial symptoms. In the first week of treatment, follow‐up was done every 3 days. Then twice a month (2 weeks apart), and an examination was done in the third month after starting the treatment. Also, a microbial culture sample was taken from the patient every 3 days. Semiquantitative microbial cultures were performed to determine the number of bacteria. According to the literature, no growth is reported as 0, rare growth as 1+, light growth as 2+, moderate growth as 3+, and heavy growth as 4+. Considering that the samples were taken from the sixth day, culture results are available from this day. Starting treatment for gram‐negative bacilli on Day 6, the culture was 3+, and this result persisted until Day 9. From the 12th day, a decrease in the bacterial load was observed, and the culture was 2+. On the 14th day, eye discharge had completely stopped, but there was slight redness. The reduction in the bacterial load indicated an appropriate response to antibiotic therapy. On the 15th day, the culture was 1+, and from the 18th day, the culture became negative and the patient was successfully treated. On the 28th day, the eye was completely normal, and nothing special was observed during the examination. The culture remained negative until the 30th day, when we continued the follow‐up to ensure the success of the treatment.

## 3 DISCUSSION

*S. paucimobilis* belonged to the genus Pseudomonas until 1977.^4^ This bacterium was initially thought to cause infections in immunocompromised individuals with long‐term catheters. Compared to other gram‐negative bacteria, the cell wall of this bacterium lacks lipopolysaccharide, and this deficiency may be the reason for the low virulence of this bacterium.^5^

Despite the patient's no history of hospitalization, the isolated bacteria showed significant antibiotic resistance. Owing to the intrinsic resistance of this bacterium to colistin (as a last‐line drug for the treatment of infections with gram‐negative bacteria), this bacterium should be treated with caution. Because bacteria can spread to hospitals and be easily colonized on medical devices such as catheters and implants.

Environmental bacteria are known to be sensitive to several antibiotics. Different studies have reported different results regarding antibiotic resistance of this bacterium. Some studies have shown that this bacterium is sensitive to piperacillin/tazobactam, carbapenems, trimethoprim‐sulfamethoxazole, and aminoglycosides. However, it is mostly resistant to penicillins, first‐generation cephalosporins, and colistin.^6^ Ozdemir et al. reported that *S. paucimobilis* is resistant to ceftazidime and cefoxitin, but Toh et al. showed that the bacterium is resistant to amikacin and fluoroquinolones.^7^

At present, antibiotics such as quinolones, carbapenems, and third‐generation cephalosporins seem to be effective in treating infections associated with *S. paucimobilis*.^8^, ^9^ However, owing to the diversity of antibiotic resistance profiles of this bacterium, individualized and appropriate antibiotic therapy should be performed in accordance with the antibiogram results.^10^ Therefore, the antibiotic resistance of this bacterium should not be underestimated. As the bacterium is slow‐growing, treatment is usually initiated before diagnosis. Therefore, this bacterium has the potential to exhibit widespread antibiotic resistance in the near future.

In ocular infections, bacteria may not be detected due to the factors such as low eye fluid volume, empirical use of antibiotics, and the fact that some bacteria are fastidious. In most laboratories, the diagnosis is based on conventional tests, and MALDI‐TOF mass spectrometry (MS) is performed only in some special laboratories. Due to these limitations, it is best to use molecular methods such as 16s rRNA gene typing to rapidly detect this type of bacteria.

## 4 NUCLEOTIDE SEQUENCE ACCESSION NO.

The sequence data have been submitted to the GenBank database under accession number OM078500.

## 5 CONCLUSIONS

There have been many reports of opportunistic infections caused by gram‐negative bacteria. To our knowledge, this is the first report of ocular eye infection with *S. paucimobilis* in Iran. Ophthalmologists and laboratory technicians should consider *S. paucimobilis* when dealing with these types of infections. *S. paucimobilis* can cause diseases in both healthy and immunocompromised individuals and should not be considered a contaminant.

## AUTHOR CONTRIBUTIONS

**Fatemeh Zeynali Kelishomi:** Investigation; writing – original draft; writing – review and editing. **Faezeh Mohammadi:** Methodology. **Mohaddeseh Khakpoor:** Investigation. **Rasoul Malekmohammadi:** Methodology. **Farhad Nikkhahi:** Conceptualization; project administration; supervision.

## CONFLICT OF INTEREST STATEMENT

All authors declare that they have no conflicts of interest in the context of this work.

## ETHICS APPROVAL AND CONSENT TO PARTICIPATE

This study was approved by the Ethics Committee of Qazvin University of Medical Sciences [approval no. IR.QUMS.REC.1401.089]. The authors confirm that the work was carried out with the ethical standards set forth in the Helsinki Declaration of 1975.

## CONSENT

Written informed consent was obtained from the patient to publish this report in accordance with the journal's patient consent policy.

## Data Availability

The datasets used and/or analyzed during the current study are available from the corresponding author on reasonable request.
